# Carbon Nanotube Immunotoxicity in Alveolar Epithelial Type II Cells Is Mediated by Physical Contact-Independent Cell–Cell Interaction with Macrophages as Demonstrated in an Optimized Air–Liquid Interface (ALI) Coculture Model

**DOI:** 10.3390/nano14151273

**Published:** 2024-07-29

**Authors:** Brijesh Yadav, Jagjit S. Yadav

**Affiliations:** Pulmonary Pathogenesis and Immunotoxicology Laboratory, Department of Environmental and Public Health Sciences, University of Cincinnati College of Medicine, Cincinnati, OH 45267, USA; brijeshsgpgims@gmail.com

**Keywords:** carbon nanotube, MWCNT, inhalation toxicity, immunotoxicity, air–liquid interface, coculture, alveolar epithelial cells, alveolar macrophages

## Abstract

There is a need for the assessment of respiratory hazard potential and mode of action of carbon nanotubes (CNTs) before their approval for technological or medical applications. In CNT-exposed lungs, both alveolar macrophages (MФs), which phagocytose CNTs, and alveolar epithelial type II cells (AECII cells), which show tissue injury, are impacted but cell–cell interactions between them and the impacted mechanisms are unclear. To investigate this, we first optimized an air–liquid interface (ALI) transwell coculture of human AECII cell line A549 (upper chamber) and human monocyte cell line THP-1 derived macrophages (lower chamber) in a 12-well culture by exposing macrophages to CNTs at varying doses (5–60 ng/well) for 12–48 h and measuring the epithelial response markers for cell differentiation/maturation (proSP-C), proliferation (Ki-67), and inflammation (IL-1β). In optimal ALI epithelial-macrophage coculture (3:1 ratio), expression of Ki-67 in AECII cells showed dose dependence, peaking at 15 ng/well CNT dose; the Ki-67 and IL-1β responses were detectable within 12 h, peaking at 24–36 h in a time-course. Using the optimized ALI transwell coculture set up with and without macrophages, we demonstrated that direct interaction between CNTs and MФs, but not a physical cell–cell contact between MФ and AECII cells, was essential for inducing immunotoxicity (proliferative and inflammatory responses) in the AECII cells.

## 1. Introduction

Engineered nanomaterials (ENMs) possess unique physicochemical properties, and are extensively used in various sectors, including medical (e.g., as a drug-delivering agent for treating cancers and drug-resistant bacteria [1]), agriculture (e.g., as fertilizers and pesticides [2]), electronics (e.g., as semiconductors, capacitors, cables, and biosensors), among several other applications [3]. Exposure to nanomaterials may induce or exacerbate acute and chronic respiratory conditions and illnesses [4,5]. Among ENMs, carbon-based nanomaterials are a major group, which exists in various forms such as nanotubes, nanofibers, fullerenes, and nanorods, among others. In particular, carbon nanotubes (CNTs) are high-volume carbon NMs, designed either as single-walled CNTs (SWCNTs) or multi-walled CNTs (MWCNTs) by wrapping layer(s) of graphite over a honeycomb lattice skeleton as a fibrous tube-shaped structure [6]. Between these two CNT forms, MWCNTs have been dominating the market. The latest trend towards incorporation of CNTs in composite materials has further enhanced their production and use in various applications. The global market size of CNTs has shown tremendous growth. According to the estimates and projections by Fortune Business Insights for the period 2023–2033, the CNT market size was USD 6.3 million in 2023 and is projected to grow at a compound annual growth rate (CAGR) of 13.3% to reach USD 18.67 billion by the year 2032 [7]. Other estimates are projecting even higher CAGR (16.1%) and estimating it to reach USD 22.3 billion by 2032 [8].

Carbon nanotubes have been shown to be toxic in biological systems because of their asbestos-like properties, such as high aspect ratio, insolubility, and non-degradability [9,10]. The common routes of human exposure to CNTs are inhalation, dermal, intravenous, and oral. CNTs have the propensity to become airborne during manufacturing, handling, and product life cycle, raising a high probability of human inhalational exposure to this material [9,11] as an occupational and environmental hazard.

Inhalation of airborne CNTs often manifests in the form of lung toxicity but may even cause systemic toxicity [12,13]. Studies by us and others have shown that CNT-induced toxicity in the lung involves inflammation, oxidative stress, DNA damage, lysosomal degradation, and mitochondria dysfunction, causing local pathological effects (granuloma, hyperplasia, fibrosis, mesothelioma) [6,14]. In the context of systemic effects, we observed inhaled MWCNTs’ effects on gut, oral, and nasal mucosa, causing microbiota dysbiosis and differential modulation of immune response genes [12,15].

Considering their adverse biological effects, screening of these nanomaterials for safety and toxicity is the prime industrial need before their approval for practical applications. Use of animal models is considered the gold standard for such toxicity screenings. However, this approach is economically expensive and ethically undesirable. Therefore, in vitro screening techniques are considered a desirable alternative. Besides screening for toxicity, investigating the nature and mechanism(s) of inhalation toxicity of airborne CNTs is crucial in understanding the respiratory health risks through occupational and environmental exposures. While the majority of such mechanistic toxicity studies are also conducted using animal models, there is a dire need for developing mechanism-driven in vitro toxicity models. Such in vitro models will help elucidate cell–cell interactions and mechanistic targets and responses and may also help design improved mechanism-inspired toxicity screening.

For predictive in vitro screening, the majority of the strategies have primarily relied on the use of in vitro submerged monocultures of lung cells, which may not reflect the in vivo context. Moreover, the threshold dose for in vitro toxicity of CNTs depends on the cell type exposed and requires optimization. For instance, murine bone marrow derived dendritic cells showed apparent apoptosis at a concentration range 3–30 μg/mL, whereas murine macrophage cell line Raw264.7 showed no obvious toxicity even at 300 μg/mL [16]. Considering these limitations, efforts are ongoing to optimize alternative in vitro toxicity testing models based on an air–liquid interface (ALI) platform and coculture of more than one cell type. Considering that lung toxicity involves interaction of CNTs with epithelial and immune cells, a coculture of these cell types may be desirable for a more realistic in vitro screening platform for assessing their respiratory toxicity hazard potential [17,18].

Due to the need to understand the toxicity mechanisms of CNTs, our previous mouse exposure studies have shown that multi-walled CNTs (MWCNTs) cause lung immunotoxicity manifested as alveolar inflammation, epithelial hyperplasia, and fibrosis [10,14,19]. Alveolar macrophages (MФs) are the lung resident phagocytic cells populating the bronchoalveolar space which maintain the local immunological homeostasis and clearance of the debris [20,21]. In our short-term exposure mouse model, MWCNT-exposed mouse lungs have shown that alveolar MФs serve as the effector cells in causing the acute inflammatory response [22]. Persistent stimulation of MФs is also known to damage the organ [23]. In our subchronic (28-day) and chronic (56-day) exposure mouse models of MWCNT immunotoxicity, alveolar epithelial type II cells (AECII cells) showed inflammatory and proliferation (hyperplasia) response [14]; the analyses had shown induction of IL-1β, pro-surfactant protein-C (Pro-SP-C), and cell proliferation (Ki-67 expression) in AECII cells and granuloma formation in lung parenchyma [14].

Alveolar epithelial cells (AECs) make up the primary lining of the lung alveolar sac and are required for mediating gaseous exchange, reducing surface tension, and defending from microbial infections, among several other functions [24]. Dysfunctional AECs have been associated with acute and chronic lung diseases such as acute distress syndrome, chronic obstructive pulmonary disease, idiopathic pulmonary fibrosis, and pneumonia [25]. Inhalation exposure to environmental toxicants such as CNTs causing AEC dysfunction, may lead to the development or exacerbation of these lung diseases. However, there is no clear understanding of the nature of cell–cell interactions leading to in vivo immunotoxicity response in the CNT-exposed lungs. For instance, the nature and mode of interactions between epithelial cells and MФs in the exposed alveoli are poorly understood. It is unclear whether a direct contact between alveolar MФs and AECs is required or whether an indirect remote contact causes the inflammatory and proliferative/hyperplasia response in AECs observed in our subchronic animal model studies.

In the current study, we tested our hypothesis that CNT-stimulated MФs induce an inflammatory and proliferative/hyperplasia response in AECII cells through a contact-independent mechanism. To accomplish this, we optimized an ALI coculture model based on human AECII cell line and human macrophage cell line. The developed ALI epithelial-macrophage coculture model will facilitate animal-free mechanistic investigations such as those to delineate gene x environment interactions. Also, this mechanism-inspired alternative in vitro testing strategy would permit rapid predictive toxicity screening so a more detailed follow up in vivo evaluation in animal models of only selected ‘carbon nanotubes of concern’ is required.

## 2. Material and Methods

### 2.1. Chemical and Reagents

MWCNT material (Baytubes^®^) was obtained from Bayer Material Science (Leverkusen, Germany) as a dry bulk powder. Pluronic F-127 was purchased from Sigma (St. Louis, MO, USA). For the cell culture work, human alveolar epithelial type II cell line A549 and human monocyte cell line THP-1 (American Type Culture Collection, Manassas, VA, USA), RPMI basal medium, lipopolysaccharide/LPS, and Phorbol 12-myristate 13-acetate/PMA (Sigma, St. Louis, MO, USA), fetal bovine serum/FBS (Gibco, Grandland, NY, USA), antibiotics (Fisher Scientific, Hampton, NH, USA), and trypsin-EDTA (Invitrogen, Waltham, MA, USA) were obtained from the respective sources indicated.

### 2.2. CNT Preparation and Characterization

High purity MWCNT powder was suspended in 1% Pluronic F-127 solution (Sigma, St. Louis, MO, USA) to prepare a homogenous suspension as master stock (1 mg/mL), and the resulting CNT suspension was characterized using methods described in our previous report [22]. Briefly, CNTs were characterized by scanning electron microscopy (for size and morphology), Brunauer, Emmett, and Teller (BET) method (for surface area), and thermogravimetric analysis. The CNT powder suspended in 1% Pluronic F-127 medium was ultrasonicated for 2 h until uniform dispersion was formed. The suspension was centrifuged at 3200× *g* for 30 min and large aggregates were pelleted and dried to determine the exact CNT concentration in suspension. The resulting master stock suspension was assayed for the endotoxin contamination by Limulus Amoebocyte assay (Pierce, Rockford, IL, USA).

For preparing a working stock for in vitro cell treatment experiments, the master stock of MWCNT suspension was diluted appropriately in FBS-free RPMI medium.

### 2.3. In Vitro Air–Liquid Interface (ALI) Coculture Setup

#### 2.3.1. THP-1 Monocyte Cell Line Differentiation into Macrophages and Culturing

Before use in in vitro CNT treatment experiments, human THP-1 cell line was differentiated into macrophage phenotype by stimulating with LPS (100 ng/mL) and PMA (10 ng/mL) for 48 h in a T-25 culture flask. Differentiated cells were trypsinized with 0.05% trypsin-EDTA solution for 3–4 min and collected using a cell scraper after trypsin activity was inactivated with 10 mL RPMI medium containing 10% FBS. Cells were washed (2×) with 5 mL RPMI medium and the cell pellet was suspended in PBS (1 mL) followed by live cell counting based on trypan blue exclusion method.

**Figure 1:**
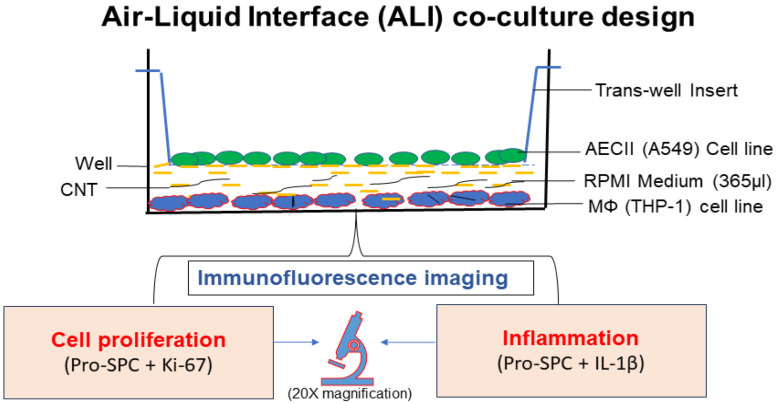
Diagrammatic representation of the air–liquid Interface (ALI) coculture model. The ALI culture was setup by placing transwell (0.4 µm pore size) inserts in individual wells of a 12-well culture cluster. Human THP-1 differentiated macrophages (15 × 10^3^ cells) were seeded in a given culture cluster well (lower compartment) and treated with CNT suspension at a defined test dose in a defined volume (315 µL) of complete RPMI medium. The transwell chamber (upper compartment) seeded with A549 alveolar epithelial type II cells (45 × 10^3^ cells) in 50 µL of complete RPMI medium was then inserted. The defined total volume of the RPMI medium (315 + 50 = 365 µL) allowed creation of an air–liquid interface for the epithelial cells in the transwell in continuity with the macrophages in the lower chamber.

For the in vitro ALI coculture experiments described below, THP-1-differentiated macrophage cells were first cultured as a monolayer in complete RPMI medium by seeding (15 × 10^3^ cells/well) in a 12-well culture plate and allowing to adhere for 12 h at 37 °C in a 5% CO_2_ incubator at 95% humidity. Triplicate wells were prepared for the following treatment groups: untreated control (RPMI medium only), vehicle control (culture treated with 1% Pluronic F-127 solution), and CNT-treated (culture treated with MWCNT dose for 48 h) (Figure 1).

#### 2.3.2. Human Alveolar Type-II Epithelial Cell Line (A549) Culture

A549 cells (45 × 10^3^) were seeded in a 0.4 µm pore size transwell insert (Corning, Rockville, MD, USA) in triplicate for each treatment group and allowed to adhere for 12 h by incubating at 37 °C in a 5% CO_2_ incubator set at 95% relative humidity.

#### 2.3.3. ALI Transwell Coculture Set Up

To set up the ALI coculture treatments, transwell inserts seeded with A549 epithelial cells (in 50 µL of RPMI medium/insert) as prepared above were transferred (as upper chamber) to individual wells (as lower chamber) of the 12-well culture cluster monolayered with adhered THP-1 macrophage cells (triplicate wells) treated with a defined concentration of CNTs (using 315 µL of RPMI medium/well). The ALI coculture cluster was then incubated for 48 h in a 5%CO_2_ incubator set at 37 °C temperature and 95% humidity. For a dose–response experiment, macrophages (lower chamber) were treated with increasing concentration of CNT material (5, 10, 15, 20, 40, and 60 ng/well) to select the optimal dose required to elicit robust inflammatory and proliferative responses in A549 cells (upper chamber). A time-course experiment was then performed using the selected effective dose to assess the dynamics of the induced cellular response in A549 cells.

A modified ALI treatment set up without macrophages in the lower chamber was used to investigate contact-independent communication between AECII cells and macrophages. This set up also allowed us to study the involvement of macrophages in the response of AECII cells to CNT.

### 2.4. Immunofluorescence Staining and Confocal Microscopy

After 48 h of co-culture, A549 cells were stained for proSP-C (lung surfactant protein-C as a cell maturation marker), IL-1β (inflammation marker), and Ki-67 (cell proliferation marker protein indicating hyperplasia effect), using specific antibodies. In brief, the cells were fixed with cold methanol for 5 min followed by permeabilization with PBS + 0.1% TritonX-100 (0.1% PBST) buffer for 10 min and subsequent non-specific site blocking with 2% Donkey serum in PBS. The cells were incubated with rabbit anti-human proSP-C (Millipore, Burlington, MA, USA), mouse anti-human IL-1β (Thermofisher, Waltham, MA, USA), and rat anti-human Ki-67 (Thermofisher, Waltham, MA, USA) antibodies for 3 h at room temperature in a humidified closed slide chamber. After washing with PBS, the cells were incubated with goat anti-rabbit IgG Dylight-594 and goat anti-mouse IgG Alexa Flour-488 conjugated secondary antibody against proSP-C and IL-1β. FITC-conjugated anti-rat IgG antibody was used for Ki-67 staining. Co-staining was performed using combinations of proSP-C and Ki-67, proSP-C, and IL-1β on separate slides. The cells were washed with PBS and stained for nucleus with DAPI for 8 min. The stained cells were mounted and analyzed using a Zeiss microscope at 20× amplification equipped with Zenblack software, version 3.9. Five adjacent focus areas were scanned and the images captured from each slide, followed by image analysis using Image-J software, version 1.53g. Fluorescence intensity of each cell was captured and total number of cells was counted based on nuclear staining. Mean fluorescence intensity (MFI) was calculated by normalizing with total number of cells (sum of total intensity from the cells per focus area/total no. of cells in the focus area). Normalized intensity values among the groups were statistically compared using ANOVA analysis with a Tukey post hoc correction test using GraphPad software version 8.0 for Windows (La Jolla, CA, USA). A *p*-value of ≤0.05 represented statistical significance.

## 3. Results

An ALI coculture model for CNT toxicity screening was successfully established (Figure 1) based on human AECII cell line A549 grown in the transwell and human THP-I cell line-derived MФs grown in the bottom well of a culture cluster as assessed based on robust detection of expression markers for AECII cell maturation (proSP-C), cell proliferation (Ki-67), and inflammation (IL-1 β).

### 3.1. Optimization of ALI Coculture Model to Screen for MWCNT Immunotoxicity Potential

#### 3.1.1. Dose-Dependence of CNT-Induced Proliferation of Human AECII Cells (A549) in the Presence of Human Macrophages (THP-1) in ALI Coculture

**Figure 2:**
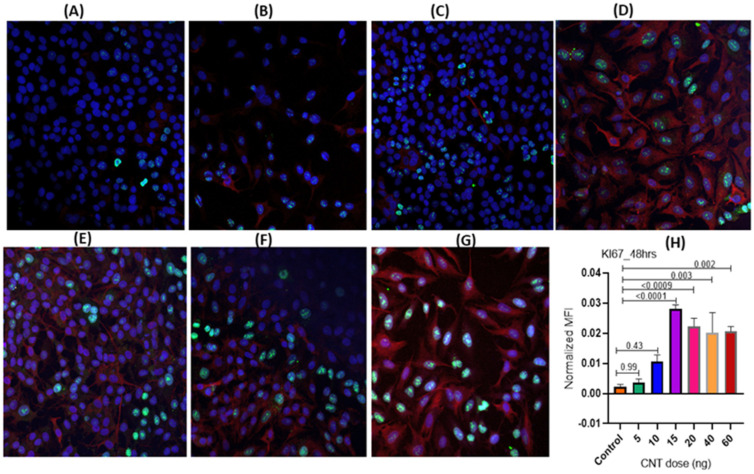
(**A**–**H**) Dose–response analysis for optimization of CNT dose in the ALI coculture model set up as described in Figure 1. The THP-1 derived macrophages seeded in individual wells in the culture cluster were treated (in triplicate) with an increasing dose of CNTs (5–60 ng/well). The cellular response in A549 cells (AECII cells) was tracked for expression of the marker for cell proliferation (Ki-67) using immunofluorescence microscopy as shown in the following figure panels corresponding to varying CNT doses: untreated control (**A**), 5 ng (**B**), 10 ng (**C**), 15 ng (**D**), 20 ng (**E**), 40 ng (**F**), 60 ng (**G**). Dose–response was assessed by plotting Ki-67 expression levels at different doses of CNT exposure (**H**). Each response marker was measured in triplicate A549 cultures, with 5 spots imaged per culture. Red and green fluorescence represented expression of proSP-C (cytoplasmic) and Ki-67 (nuclear), respectively, whereas blue fluorescence (DAPI stain) represented the nucleus. ProSP-C expression was tracked qualitatively as an index of desired cell differentiation/maturation of the AECII cells. Statistical significance was based on *p* ≤ 0.05. *p*-values indicate extent of statistical significance as compared to the control.

Using increasing doses of MWCNTs (5, 10, 15, 20, 40, and 60 ng per well) to treat MФs (lower chamber) for 48 h, we determined an effective dose of CNTs that could induce optimal levels of cell proliferation marker Ki-67 in AECII cells (transwell) showing the desired differentiation/maturation (as assessed by proSP-C expression). The 15 ng/well dose was found to be the effective dose, showing the highest induction of Ki-67 expression, although the responses were also observed at the lower doses. This selected effective dose was used for all subsequent experiments (Figure 2A–H).

#### 3.1.2. Temporal Pattern of CNT-Induced Proliferative- and Inflammatory-Responses in Human AECII Cells (A549) in the Presence of Human Macrophages (THP-1) in ALI Coculture

**Figure 3:**
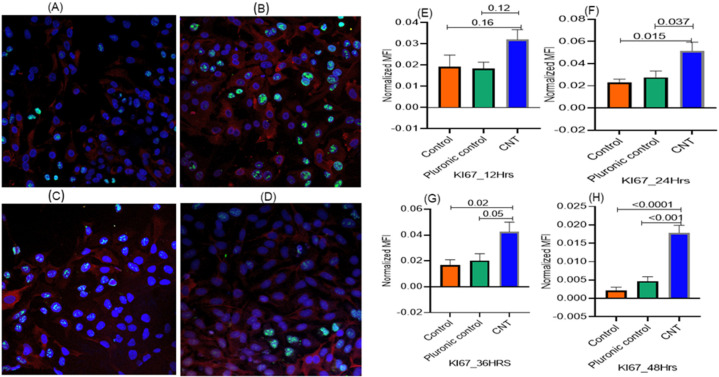
(**A**–**H**) Temporal analysis for optimization of the time required to elicit cell proliferation response to CNT treatment in alveolar epithelial cells in the ALI coculture model set up as described in Figure 1. The THP-1-derived macrophages seeded in individual wells in the culture cluster were treated with a defined dose of CNTs (15 ng/well) and including both untreated control and Pluronic F-127 (vehicle)-treated control. The response in terms of expression of the marker for cell proliferation (Ki-67) was tracked in the A549 cells (AECII cells) in the transwell; the temporal response was quantified at the following time points: 12 h (**A**,**E**), 24 h (**B**,**F**), 36 h (**C**,**G**), and 48 h (**D**,**H**). The response markers were measured in triplicate A549 cultures, with 5 spots imaged per culture. Ki-67 expression detectable within 12 h reached its maximum at 24 h. Red and green fluorescence represented expression of proSP-C (cytoplasmic) and Ki-67 (nuclear), respectively, and the blue fluorescence represented the nucleus. ProSP-C expression was tracked qualitatively as an index of desired cell differentiation/maturation of the AECII cells. Statistical significance was based on *p* ≤ 0.05.

**Figure 4:**
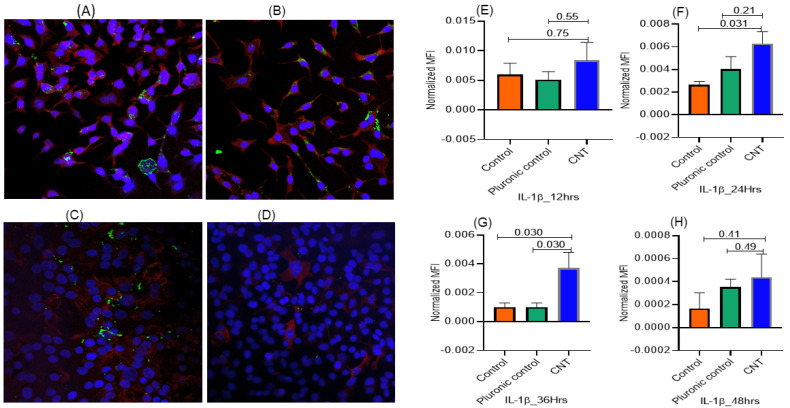
(**A**–**H**) Temporal analysis for optimization of the time required to elicit inflammatory response to CNT treatment in alveolar epithelial cells in the ALI coculture model set up as described in Figure 1. The THP-1 derived macrophages seeded in individual wells in the culture cluster were treated with a defined dose of CNTs (15 ng/well) and including both untreated control and Pluronic F-127 (vehicle)-treated control. The response in terms of expression of the marker for inflammatory response (IL-1β) was tracked in the A549 cells (AECII cells) in the transwell; the temporal response was measured at the following time points: 12 h (**A**,**E**), 24 h (**B**,**F**), 36 h (**C**,**G**), and 48 h (**D**,**H**). IL-1β expression in A549 cells detectable within 12 h reached its maximum at 24 h. The response markers were measured in triplicate A549 cultures, with 5 spots imaged per culture. Red, green, and blue fluorescence represented expression of proSP-C, IL-1β, and nucleus, respectively. ProSP-C expression was tracked qualitatively as an index of desired cell differentiation/maturation of the AECII cells. Statistical significance was based on *p* ≤ 0.05.

Using the effective CNT dose (15 ng/well) in the ALI coculture, the expression of both Ki-67 (proliferation marker) and IL-1β (inflammatory response marker) was detectable as early as 12 h of treatment. The Ki-67 expression in AECII cells reached its maximum within 24 h incubation and was significantly higher compared to untreated control and vehicle (Pluronic)-treated control. The intracellular levels of both IL-1β and Ki-67 in the AECII cells decreased after 24 h, suggesting maturation and release of IL-1β into the culture supernatant (Figure 3 and Figure 4).

### 3.2. Macrophages Found Indispensable for CNT-Induced Response (Ki-67, IL-1β) in AECII Cells

**Figure 5:**
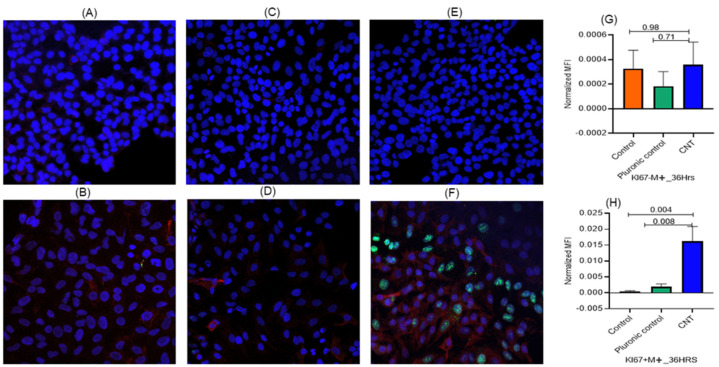
(**A**–**H**) Role of macrophages in eliciting cell proliferation response to CNT treatment in alveolar epithelial cells in the ALI coculture model set up as described in Figure 1 with modification in terms of macrophages. The ALI system was set up with or without macrophages in the lower chamber and with A549 cells (AECII cells) in the upper chamber (transwell) and run for 36 h using a defined dose of CNTs (15 ng/well) and including both untreated control and Pluronic F-127 (vehicle)-treated control. The response was tracked in terms of expression of the marker for cell proliferation (Ki-67) in the A549 cells (AECII cells) in the transwell. The response markers were measured in triplicate A549 cultures, with 5 spots imaged per culture. Red, green, and blue fluorescence represented expression of proSP-C, Ki-67, and nucleus, respectively. ProSP-C expression was tracked qualitatively as an index of desired cell differentiation/maturation of the AECII cells. The role of macrophages was assessed based on the Ki-67 expression levels in presence or absence of macrophages in the lower chamber. Ki-67 expression in epithelial cells was detected only when macrophages were included (lower figure (**B**,**D**,**F**,**H**)) and was not detected in absence of macrophages (upper figure (**A**,**C**,**E**,**G**)) in the ALI set up. This suggested an effector role of macrophages in eliciting epithelial response to CNTs in a physical contact-independent manner via diffusible soluble mediator(s). Statistical significance was based on *p* ≤ 0.05.

**Figure 6:**
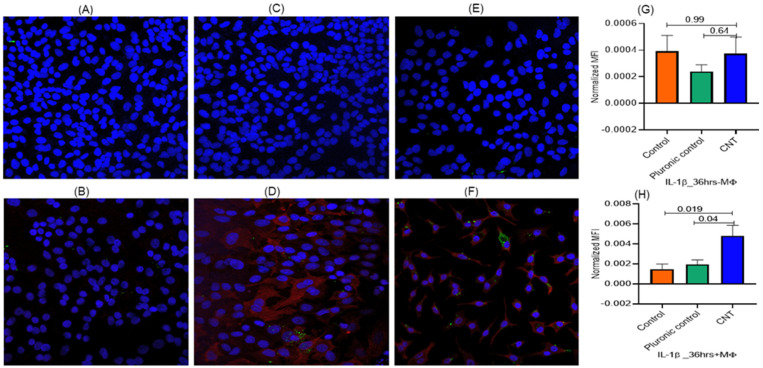
(**A**–**H**) Role of macrophages in eliciting inflammatory response to CNT treatment in alveolar epithelial cells in the ALI coculture model set up as described in Figure 1 with modification in terms of macrophages. The ALI system was set up with and without macrophages in the lower chamber and with A549 cells (AECII cells) in the upper chamber (transwell) for 36 h using a defined dose of CNTs (15 ng/well) and including both untreated control and Pluronic F-127 (vehicle)-treated control. The response was tracked in terms of expression of the marker for inflammatory response (IL-1β) in the A549 cells (AECII cells) in the transwell. The response markers were measured in triplicate A549 cultures, with 5 spots imaged per culture. Red, green, and blue fluorescence represented expression of proSP-C, IL-1β, and nucleus, respectively. ProSP-C expression was tracked qualitatively as an index of desired cell differentiation/maturation of the AECII cells. The role of macrophages was assessed based on the IL-1β expression levels in the presence or absence of macrophages in the lower chamber. IL-1β expression in epithelial cells was detected only when macrophages were included (lower figure (**B**,**D**,**F**,**H**)) and was not detected in absence of macrophages (upper figure (**A**,**C**,**E**,**G**)) in the ALI set up. This suggested an effector role of macrophages in eliciting epithelial inflammation response to CNTs in a physical contact-independent manner via diffusible soluble mediator(s). Statistical significance was based on *p* ≤ 0.05.

To check whether Ki-67 and IL-1β expressions were independent of the MФs, we set up ALI cultures of AECs cells in presence or absence of THP-1 cells and using the effective CNT dose (15 ng) treatment for 36 h. The results showed that the expression of both the response markers (Ki-67 and IL-1β) in AECII cells essentially required the presence of MФs as a coculture (Figure 5 and Figure 6).

## 4. Discussion

The use of animal models has been considered the gold standard for evaluation of inhalation toxicity potential of hazardous materials, including carbon nanomaterials, but this option is expensive and associated with ethical concerns. These caveats have spurred interest in evaluating the use of in vitro cell culture-models based on lung cells. The majority of the studies in this direction have focused on monocultures of epithelial cells or another cell type grown as submerged monolayer culture models, which often fail to mimic the lung response [26,27]. In the current study, we rationalized that the in vitro air–liquid interface (ALI) cell culture model based on lung cells, particularly as a coculture of more than one cell type, is physiologically more realistic to mimic in vivo lung cell interactions and offers breadth to study the inhalation toxicity testing and underlying mode of action with higher sensitivity [28,29].

The human alveolar epithelial type II cell line A549 consistently recapitulates the property and response of lung alveolar epithelium, such as pro-surfactant protein secretion, inflammation, metabolic activity, and has the potential to be used for developing an in vitro culture model [30]. On the other hand, alveolar MФs, the resident leukocytes in airways, remain in close crosstalk with the alveolar epithelial cells and environmental inhalants. They primarily phagocytose exogenous particulates and endogenous debris and clear the airways by various mechanisms, such as respiratory burst, secreting mucin, and recruiting other mononuclear cells, among others [21,31]. The lung MФs also have profibrotic activity mediating epithelial injury by secreting fibrogenic molecules. Collectively, this emphasizes the potential of coculturing AECII cells and MФs to realistically mimic the lung alveolar microenvironment.

We rationalized that the ALI platform could be a preferred model for the coculturing, particularly to dissect the complex crosstalk of alveolar MФs with AECs and exogenous material [32]. Additionally, ALI requires a minimum amount of growth media (partially soaking the epithelial cells in the transwell culture format), thereby mimicking the airway epithelial architecture and enhancing probability of realistic interaction with the inhaled particles or physiological inducers, thus increasing the sensitivity of the assay [33]. This is consistent with earlier reports showing greater sensitivity of the ALI format over the submerged format in monocultures of alveolar epithelial cells as a model of toxicity assessment of metal-based ENMs [28,34].

The current study was inspired by our preceding mouse model studies on lung immunotoxicity of MWCNTs, wherein we observed both inflammation (IL-1β induction) and hyperplasia in AECII cells and detection of MФs in proximity to the AECII cells in the exposed lungs [14]. This led to a mechanism-centric design and optimization of an ALI epithelial-macrophage coculture model in this study for assessing the immunotoxicity potential of CNTs.

ALI co-culture optimizations were based on cellular expression of inflammatory molecule IL-1β, cell proliferation marker Ki-67 (indicating hyperplasia), and lung defense surfactant protein pro-SP-C in the AECII cells. In dose–response analysis, cellular expression of Ki-67 in AECII cells was increased with the increase in MWCNT dose, reaching its peak at the 15 ng/well dose, followed by a decrease with the increase in CNT doses. This decline at higher doses might be due to cytotoxicity and/or cell death.

In time-course analysis, we observed induction of proIL-1β and Ki-67 gene expression in AECII cells within 12 h of CNT treatment of MФs. A decline in the intracellular expression level of IL-1β in time points beyond 24 h incubation might be because of a cleavage of proIL-1β into IL-1β and secretion in the culture supernatant due to activation of a multiprotein complex inflammasome [35,36]. IL-1β is a proinflammatory cytokine that is known to induce epithelial to mesenchymal transition (EMT) in A549 cell line during chronic inflammation and ultimately leads to pulmonary fibrosis and progression of cancer [37]. We also observed detection of cell proliferation marker Ki-67 within 12 h of stimulation, which peaked in 24–36 h, indicating hyperplasia effect.

Similar to dose response, the temporal expression of Ki-67 increased up until 24 h of incubation, followed by a decrease, which may be due to induction of apoptosis, as reported in other studies [38,39]. AECII cells expressed the surfactant protein proSP-C within 12 h of MWCNT stimulation of MФs, possibly as a response to mitigate the toxicity of CNTs. In an earlier study, incubation of proSP-C with mesoporous CNTs was associated with reduced inflammation and cytotoxicity [40]. In our recent in vivo study based on a MWCNT subchronic exposure mouse model, we observed that prolonged duration (28 days) of exposure led to decreased expression of pathogen-uptake receptors on alveolar macrophages, particularly macrophage scavenger receptors class A type 1 (Msr1) and type 2 (Msr2), phosphatidyl-serine receptor (Ptdsr), and CD163. Additionally, the MWCNT-treated mice showed more polarized lung macrophages with bias toward M2b lineages. This suggested a macrophage conversion towards more immunosuppressive M2 lineage. In the current in vitro ALI coculture system, a diminished IL-1β expression after 24 h of treatment may be due to macrophage polarization towards M2 lineages [41]. Another possibility may be that the stimulated macrophage cells start expressing apoptosis mediators causing cell death, as Ki-67 expression was significantly decreased after 24 h of treatment.

In the context of macrophage polarization, there exist heterogenous lineages of macrophages that largely remain plastic dictated by the prevailing stimulus and milieu. Broadly, there are two common lineages, the classically activated macrophage (M1) and selectively activated alternate type 2 macrophage (M2). M1 macrophages are pro-inflammatory in nature and secrete pro-inflammatory cytokines (such as IL-6, TNF-α) and reactive oxygen species for the clearance of pathogens and other stimuli [42]. This may aggravate the immune response, leading to lung injury and exacerbation of disease. The M2 macrophages secrete anti-inflammatory molecules, including resistin-like-α, IL-10, Arginase-1, and mannose receptor C type 1 (MRC1), which help in resolution of inflammation and remodeling of injured lung tissue and tumor progression [43]. During extended exposures, M1 macrophages become polarized towards M2 lineage resulting in immunosuppression and poor pathogen clearance and higher infection [41].

Importantly, the results demonstrated a remote communication between AECII cells (cultured in the transwell) and THP-1-derived MФs (cultured with CNTs in the bottom well) in this ALI coculture model since the MФs were not in direct physical contact with the AECII cells. Notably, this contact-independent communication between AECII cells and MФs was confirmed using the ALI treatment design lacking macrophages, wherein no inflammatory and cell proliferative responses were induced by CNTs (present in lower chamber) in the AECII cells (present in transwell chamber). These observations confirmed remote signaling between these two cell types, possibly mediated by diffusible soluble factors released from CNT-activated MФs, leading to activation of AECII cells (measured in terms of IL-1β, Ki-67, and pro-SP-C expression). In this context, our previous in vitro and in vivo [22] experiments have shown that MWCNTs induce MyD88-dependent expression of proinflammatory soluble mediators (IL-1β and TNF-α) in the alveolar MФ cells. Briefly, we had observed [22] detectable cytokine induction beginning 6 h post-treatment and a linear increase in levels of IL-1β (0–300 pg/mL cell lysate) and TNFα (0–100 pg/mL) in monocultures of an alveolar MФ cell line treated for 24 h with increasing doses of the same MWCNT material as used in this study.

In macrophage–CNT interactions, MФs phagocytose CNTs and release proteases and reactive oxygen species (ROS) in an attempt to digest this fibrous material. However, due to the CNT material’s resistance to degradation, MФs persistently keep secreting the ROS and cytokines, which may have remotely impacted AECII cell layer in the upper chamber (transwell) in the ALI coculture [44]. Targeted further investigations will help identify such diffusible mediators generated from macrophages that are necessary for the induction of inflammatory and proliferative response in AECII cells [44]. Nonetheless, this contact-independent interaction via diffusible mediators may be in addition to the contact-dependent interaction in the in vivo scenario. For instance, in vitro LPS-induced cocultures wherein macrophages were cultured in direct contact with alveolar epithelial cells elicited an inflammatory cytokine response in the latter [45]. Importantly, the current study unveils that the in vivo CNT exposure scenario involves alveolar MФs mediating alveolar epithelial cell inflammation and proliferation by remotely secreting proinflammatory soluble factors in a contact-independent manner.

**Limitations of study**: While the current in vitro study has unambiguously demonstrated a direct contact-independent mechanism of alveolar epithelial cell activation by the CNT-activated MФs, identifying specific signaling pathways involved could have added further dimension to the underlying mechanisms. Considering our previous study, wherein we demonstrated MYD88-dependent induction of proinflammatory cytokines (IL-1β and TNF-α) from MФs after stimulation with CNTs [22], tracking diffusible soluble mediators in the treated macrophage culture supernatants in the ALI model could have expanded information on the induced inflammatory milieu. Besides Ki-67 and IL-1β, other markers of proliferation/hyperplasia and inflammation could be measured for more detailed characterization of the established ALI system. Additionally, delivery of aerosolized CNTs in the ALI culture setup may better mimic the real-world in vivo respiratory exposure scenario. Nonetheless, the current study has established a mechanism-inspired 3D ALI coculture model with a promising potential for adapting this model for in vitro high throughput testing of CNTs and other carbon nanomaterial forms, thereby circumventing the need for animal models of toxicity.

## 5. Conclusions

This study has led to in vivo mechanism-inspired design and optimization of an ALI epithelial-macrophage coculture model for immunotoxicity screening of CNTs. The optimal testing conditions include exposure to 15 ng of the test CNT material for 12 h to induce measurable markers of immunotoxicity in AECII cells in terms of inflammatory and cell proliferation response. To our knowledge, this is the first experimental demonstration of a physical contact-independent mechanism of MWCNT-induced activation of alveolar epithelial cells by macrophages.

## Data Availability

All data have been included in the manuscript.

## Abbreviations

CNT, carbon nanotube; MWCNT, multi-walled carbon nanotube; ALI, air–liquid interface; MФs, macrophages; AECII cells, alveolar epithelial type II cells; proSP-C, pro-surfactant protein C.
